# Comprehensive hemocompatibility analysis on the application of diamond-like carbon to ePTFE artificial vascular prosthesis

**DOI:** 10.1038/s41598-023-35594-7

**Published:** 2023-05-24

**Authors:** Takashi Goyama, Yasuhiro Fujii, Genya Muraoka, Tatsuyuki Nakatani, Daiki Ousaka, Yuichi Imai, Noriaki Kuwada, Tatsunori Tsuji, Takayuki Shuku, Haruhito A. Uchida, Masahiro Nishibori, Susumu Oozawa, Shingo Kasahara

**Affiliations:** 1 Department of Cardiovascular Surgery, Okayama University Graduate School of Medicine, Dentistry and Pharmaceutical Sciences, 2-5-1 Shikata-cho, Kita-ku, Okayama, Okayama 700-8558 Japan; 2 Department of Cardiovascular Surgery, Okayama University Faculty of Medicine, Dentistry and Pharmaceutical Sciences, 2-5-1 Shikata-cho, Kita-ku, Okayama, Okayama 700-8558 Japan; 3 Institute of Frontier Science and Technology, Okayama University of Science, 1-1 Ridai-cho, Kita-ku, Okayama, Okayama 700-0005 Japan; 4 Department of Pharmacology, Okayama University Faculty of Medicine, Dentistry and Pharmaceutical Sciences, 2-5-1 Shikata-cho, Kita-ku, Okayama, Okayama 700-8558 Japan; 5 Department of Cardiovascular Surgery, Kawasaki Medical Hospital, 577 Matsushima, Kurashiki, Okayama 701-0192 Japan; 6 Department of Civil Engineering, Okayama University Graduate School of Environmental and Life Science, 3-1-1 Tsushima naka, Kita-ku, Okayama, Okayama 700-8530 Japan; 7 Department of Chronic Kidney Disease and Cardiovascular Disease, Okayama University Faculty of Medicine, Dentistry and Pharmaceutical Sciences, 2-5-1 Shikata-cho, Kita-ku, Okayama, Okayama 700-8558 Japan; 8 Department of Translational Research and Drug Development, Okayama University Faculty of Medicine, Dentistry and Pharmaceutical Sciences, 2-5-1 Shikata-cho, Kita-ku, Okayama, Okayama 700-8558 Japan; 9 Division of Medical Safety Management, Safety Management Facility, Okayama University Hospital, 2-5-1 Shikata-cho, Kita-ku, Okayama, Okayama 700-8558 Japan

**Keywords:** Materials science, Engineering, Biomedical engineering, Cardiac device therapy

## Abstract

The aim of this study was to obtain comprehensive data regarding the hemocompatibility of diamond-like carbon (DLC)-coated expanded polytetrafluoroethylene (ePTFE). DLC increased the hydrophilicity and smoothened the surface and fibrillar structure, respectively, of the ePTFE. DLC-coated ePTFE had more albumin and fibrinogen adsorption and less platelet adhesion than uncoated ePTFE. There were scarce red cell attachments in in vitro human and in vivo animal (rat and swine) whole blood contact tests in both DLC-coated and uncoated ePTFE. DLC-coated ePTFE had a similar but marginally thicker band movement than uncoated-ePTFE with SDS-PAGE after human whole blood contact test. In addition, survival studies of aortic graft replacement in rats (1.5 mm graft) and arteriovenous shunt in goats (4 mm graft) were performed to compare the patency and clot formation between DLC-coated and uncoated ePTFE grafts. Comparable patency was observed in both animal models. However, clots were observed in the luminal surface of the patent 1.5 mm DLC-coated ePTFE grafts, but not in that of uncoated ePTFE grafts. In conclusions, hemocompatibility of DLC-coated ePTFE was high and comparable to that of uncoated ePTFE. However, it failed to improve the hemocompatibility of 1.5 mm ePTFE graft probably because increased fibrinogen adsorption canceled the other beneficial effects of DLC.

## Introduction

Recently, expanded polytetrafluoroethylene (ePTFE) has been the predominant fabric used for artificial vascular grafts as a small arterial bypass and arteriovenous graft (AVG) for hemodialysis. However, low patency rates due to thrombosis and neointima formation are still important concerns for these specific applications^1^.

Diamond-like carbon (DLC) film is an amorphous carbon-based film containing intermingled *sp*^3^ and *sp*^2^ carbon–carbon bonds, and is known for its high biocompatibility, infinitesimal cellular cytotoxicity, hardness, low friction coefficients, chemical inertness, and smooth surface finish^2–5^. DLC film is mainly used in the industry as a protective membrane for materials such as automobile components and machine parts. However, it has also shown promising features as a coating material for blood-contacting applications due to its superior mechanical properties and hemocompatibility^6–8^ owing to low platelet adhesion^9^ and decreased macrophage activation^10,11^. The application of DLC coating to blood-contacting medical devices, such as vascular stents^9,12^ and artificial heart diaphragms^13^ has been attempted earlier. However, to our knowledge, no study has suggested coating DLC on the internal surface of small diameter, long resin tubes, such as vascular grafts and catheters, due to difficulties in placing the film on the internal surface of a resin tube. However, we have developed a novel, successful technique for DLC coating on the internal surface of tubular resins and invented DLC-coated ePTFE grafts^14^.

The purpose of this study was to obtain a comprehensive understanding regarding the hemocompatibility of this newly developed DLC-coated ePTFE vascular graft through several in vitro and in vivo tests, including animal survival tests with aortic replacement using a 1.5 mm vascular graft in rats (small arterial bypass model) and AVG using a 4 mm vascular graft in goats (AVG model).

## Results

### Appearance and hydrophilicity of DLC-coated ePTFE

**Figure 1:**
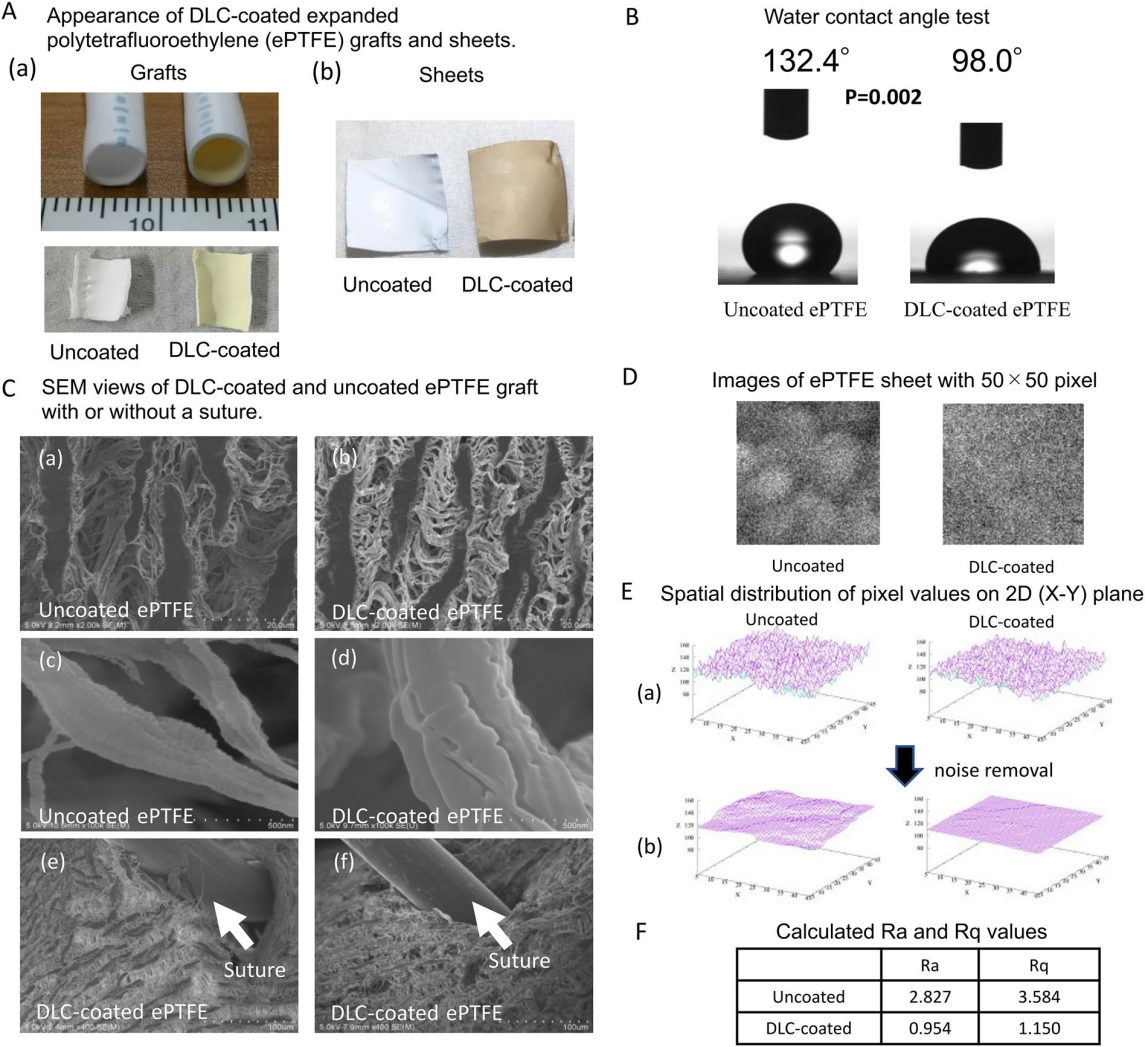
Characteristics of DLC surface. (**A**) DLC-coated ePTFE grafts and sheets. (**B**) Increased hydrophilicity of DLC-coated ePTFE. (**C**) Scanning electron microscopy (SEM) views of DLC-coated and uncoated ePTFE graft with (e–f) or without (a–d) a suture. (**D**) Images of ePTFE graft with 50 × 50 pixels. (**E**) Spatial distribution of pixel values on 2D (X–Y) plane. (**F**) Calculated Ra and Rq values.

The success of the DLC coating was confirmed using Raman spectroscopy as described previously^12^. The appearance of a DLC-coated ePTFE graft and a sheet is demonstrated in Fig. 1A. DLC added a brownish color to the ePTFE surface.

The mean water contact angle was significantly smaller (*P* = 0.002) on the DLC-coated sheets (98.0° ± 0.7°; range: 95.8°–102.2°) than on the uncoated ePTFE sheets (132.4 ± 0.7°; range: 131.6°–133.4°) (Fig. 1B). This result indicates DLC-coated ePTFE surface is more hydrophilic than uncoated ePTFE surface.

### Surface roughness of ePTFE

Scanning electron microscopy (SEM) analysis revealed that the porous structure of the ePTFE grafts were maintained regardless of the presence or absence of the DLC-coating (Fig. 1C-a,C-b). At higher magnification, small irregularities were observed on the internal surfaces of the uncoated ePTFE grafts (Fig. 1C-c); the internal surfaces of the DLC-coated ePTFE grafts were much smoother with fewer irregularities than those of the uncoated ePTFE grafts (Fig. 1C-d). In addition, no peeling was observed on the surface of the DLC-coated ePTFE grafts after anastomotic maneuver using 7–0 polypropylene suture (The needle was passed through the wall of the ePTFE graft from the outside to the inside in Fig. 1C-e, and from the inside to the outside in Fig. 1C-f). These results indicate excellent integration of ePTFE’s fibrillar structure and DLC to endure stress for forceps and needle during anastomosis procedure.

Figure 1D show images of the surface of uncoated and DLC-coated ePTFE sheets with 50 × 50 pixel. It is easy to visually understand that the DLC-coated surface is smoother than the uncoated surface. Figure 1E-a shows spatial distribution of pixel values on 2D (x–y) plane for uncoated and DLC-coated ePTFE. Both images highly fluctuated due to the noise. Therefore, noise removal was required. Figure 1E-b shows the images after noise removal using trend-filtering. Noise removal worked well, and clear structures or trends was extracted from the original pixel data. Figure 1F summarizes the results of the calculated arithmetic mean roughness (*R*_*a*_) and root-mean-square roughness (*R*_*q*_) values. Clearly, the *Ra* and *Rq* values of the DLC-coated ePTFE were smaller than those of uncoated ePTFE, which means DLC-coated surface is smoother than uncoated surface.

### Protein adsorption tests

**Figure 2:**
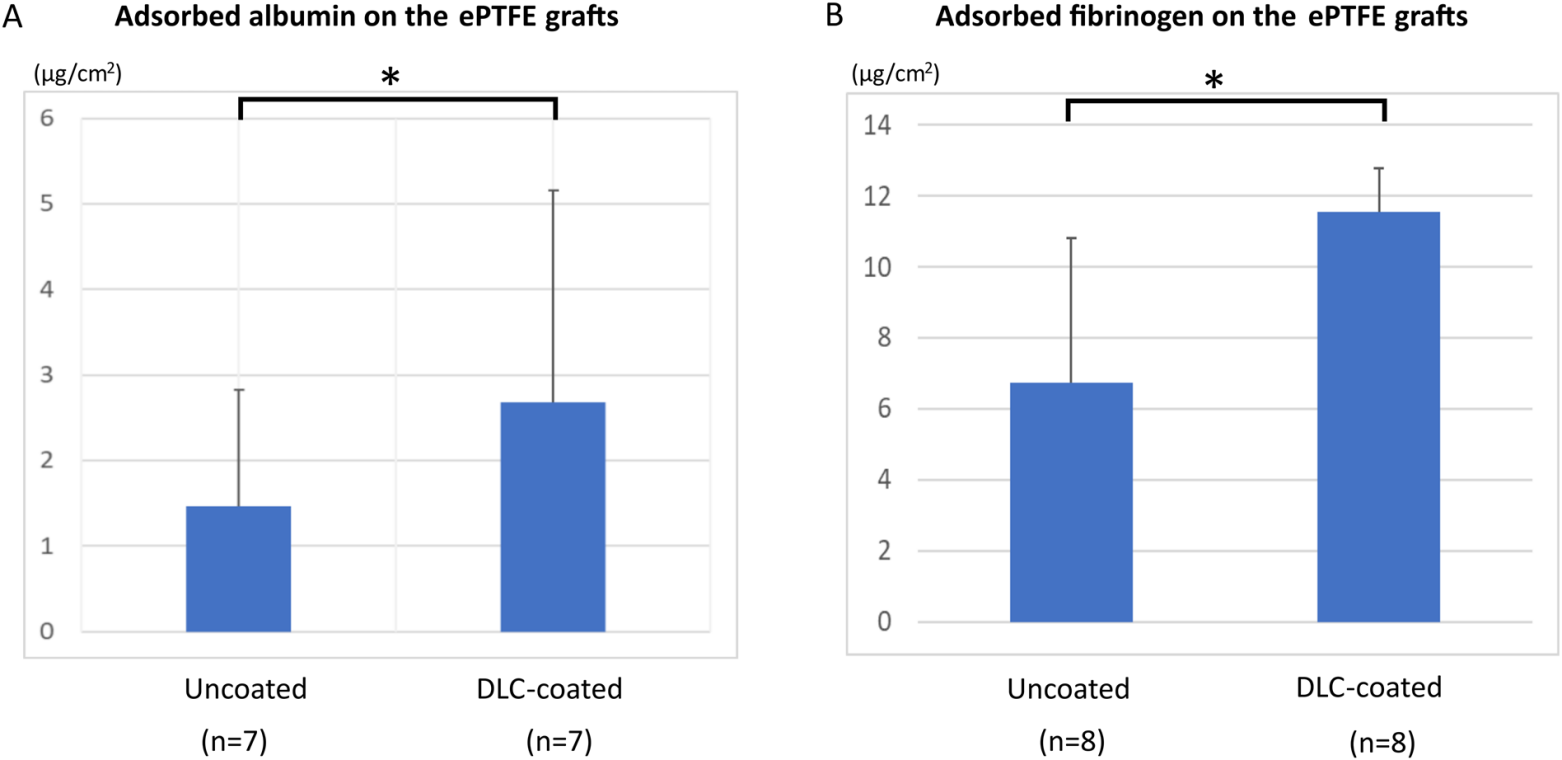
Results of albumin and fibrinogen adsorption tests in ePTFE grafts. The amount of adsorbed albumin was higher on the DLC-coated grafts than that on the uncoated grafts, respectively. Asterisks denote significant (*p* < 0.05) between the groups. Error bars represent standard deviation of the mean.

Figure 2 shows the results of protein adsorption analysis on the ePTFE grafts using human albumin and fibrinogen. In this experiment, only the internal surface of the graft was coated with DLC. Therefore, the difference between an uncoated graft and a DLC-coated graft can be only attributed to the difference of the condition on the internal surface. Even in this condition, the amount of adsorbed albumin (n = 7) was significantly higher (*P* = 0.038) on the DLC-coated grafts (2.67 ± 2.48 µg/m^2^; range: 3.42–10.08 µg/m^2^) than that on the uncoated grafts (1.47 ± 1.36 µg/m^2^; range: 0.96–4.37 µg/m^2^). Similarly, the amount of adsorbed fibrinogen (n = 8) was significantly higher (*P* = 0.011) on the DLC-coated grafts (11.54 ± 1.25 µg/m^2^; range: 10.14–13.24 µg/m^2^) than that on the uncoated grafts (6.75 ± 4.06 µg/m^2^; range: 2.14–14.31 µg/m^2^).

### Platelet adhesion test

**Figure 3:**
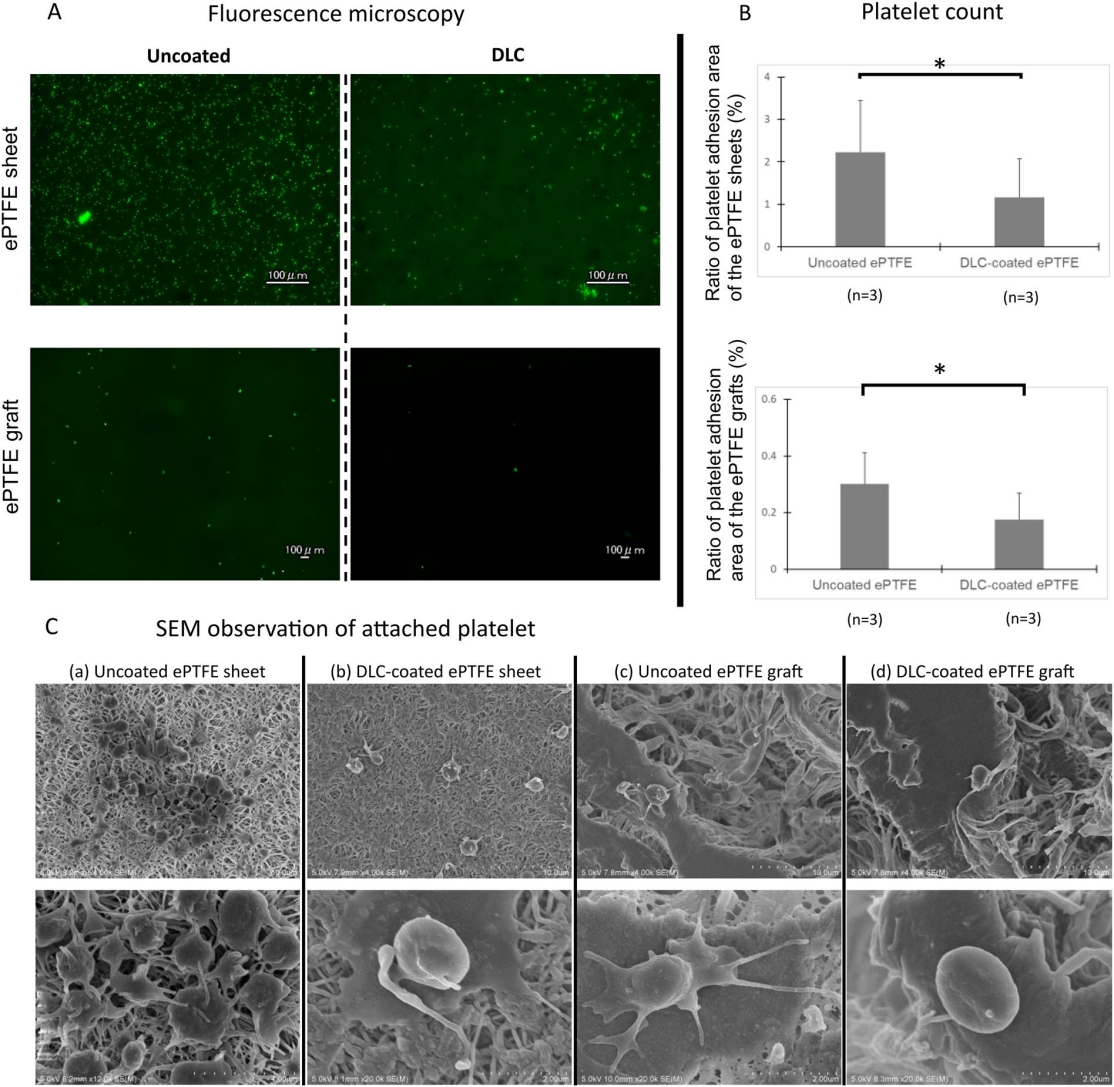
Results of platelet adhesion tests in ePTFE sheets and grafts. (**A**) The fluorescence microscope images of the surface of DLC-coated and an uncoated ePTFE graft and a sheet after platelet adhesion tests. (**B**) The results of comparison of platelet adhesion area. The ratio of platelet adhesion area was significantly smaller on the DLC-coated grafts and sheets than that on the uncoated grafts and sheets, respectively. Asterisks denote significant (*p* < 0.05) between the groups. Error bars represent standard deviation of the mean. (**C**) SEM images of attached platelets.

Figure 3 shows the results of the platelet adhesion test. Adherent platelets were much sparser on the grafts (Fig. 3A, lower row) than those on the sheets (Fig. 3A, upper row). The average ratio of platelet adhesion area (30 fields of view from 3 samples in each group) was significantly smaller (*P* = 0.015) on DLC-coated sheets (1.17 ± 0.91%; range: 0.37–4%) than that on uncoated sheets (2.22 ± 1.22%; range: 0.16–3.40%). Similarly, the average ratio of platelet adhesion area (30 fields of from 3 samples in each group) was significantly smaller (*P* = 0.003) on DLC-coated grafts (0.175 ± 0.093%; range: 0.040–0.387%) than that on uncoated grafts (0.302 ± 0.109%; range: 0.141–0.557%) (Fig. 3B). In addition, SEM revealed that platelets on the uncoated ePTFE sheets and grafts had more pseudopodia and cluster (Fig. 3C-a,C-c) than those on DLC-coated sheets and grafts. Adhered platelets were inclined to disperse and maintain their round shape on the DLC-coated sheets and grafts (Fig. 3C-b,C-d).

### In vitro human whole blood contact test (SDS-PAGE and Immunofluorescent staining of fibrin)

**Figure 4:**
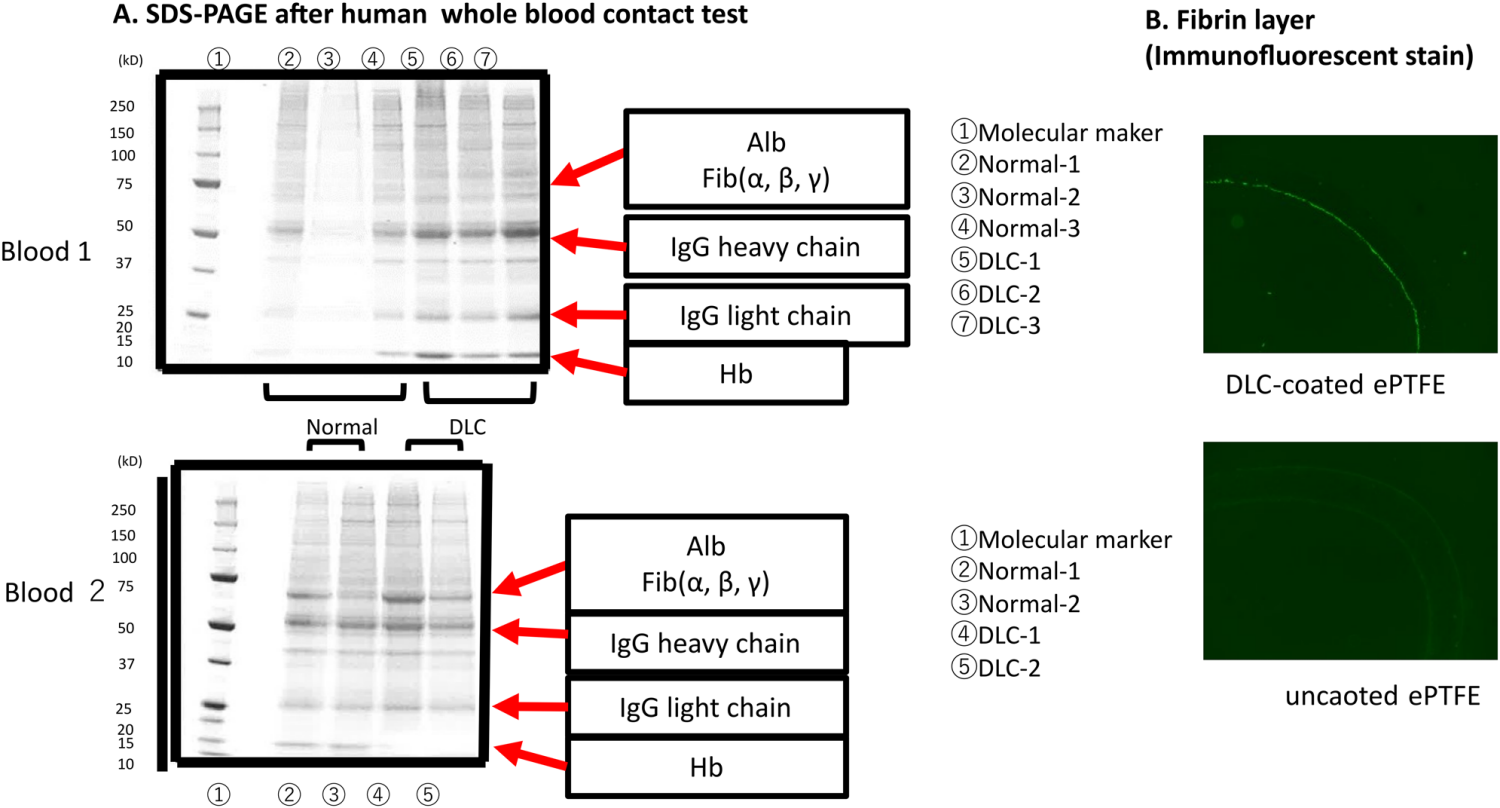
Sodium dodecyl sulphate–polyacrylamide gel electrophoresis analysis and immunofluorescent staining of fibrin after whole human blood contact test. (**A**) Both types of grafts had a similar band movement with whole blood from 2 different healthy persons. However, the bands corresponding to albumin, fibrinogen, heavy IgG, and light IgG tended to be clearer in DLC-coated grafts than those in uncoated grafts. (**B**) There was a thin fibrin layer on the inner surface of DLC-coated grafts. Alb, albumin; Fib, fibrinogen; IgG, immunoglobulin G, Hb, hemoglobin.

Figure 4 shows the results of SDS-PAGE analysis (original figures are provided as Supplementary Figure S1). Both types of grafts had a similar band movement with whole blood from 2 different healthy persons. However, the bands corresponding to albumin, fibrinogen, heavy IgG, and light IgG tended to be clearer in DLC-coated grafts than those in uncoated grafts (Fig. 4A). There was a thin fibrin layer on the inner surface of DLC-coated grafts (Fig. 4B).

### In vivo whole blood contact tests

#### Rat whole blood contact test (Macroscopic finding and immunofluorescent staining of fibrin)

**Figure 5:**
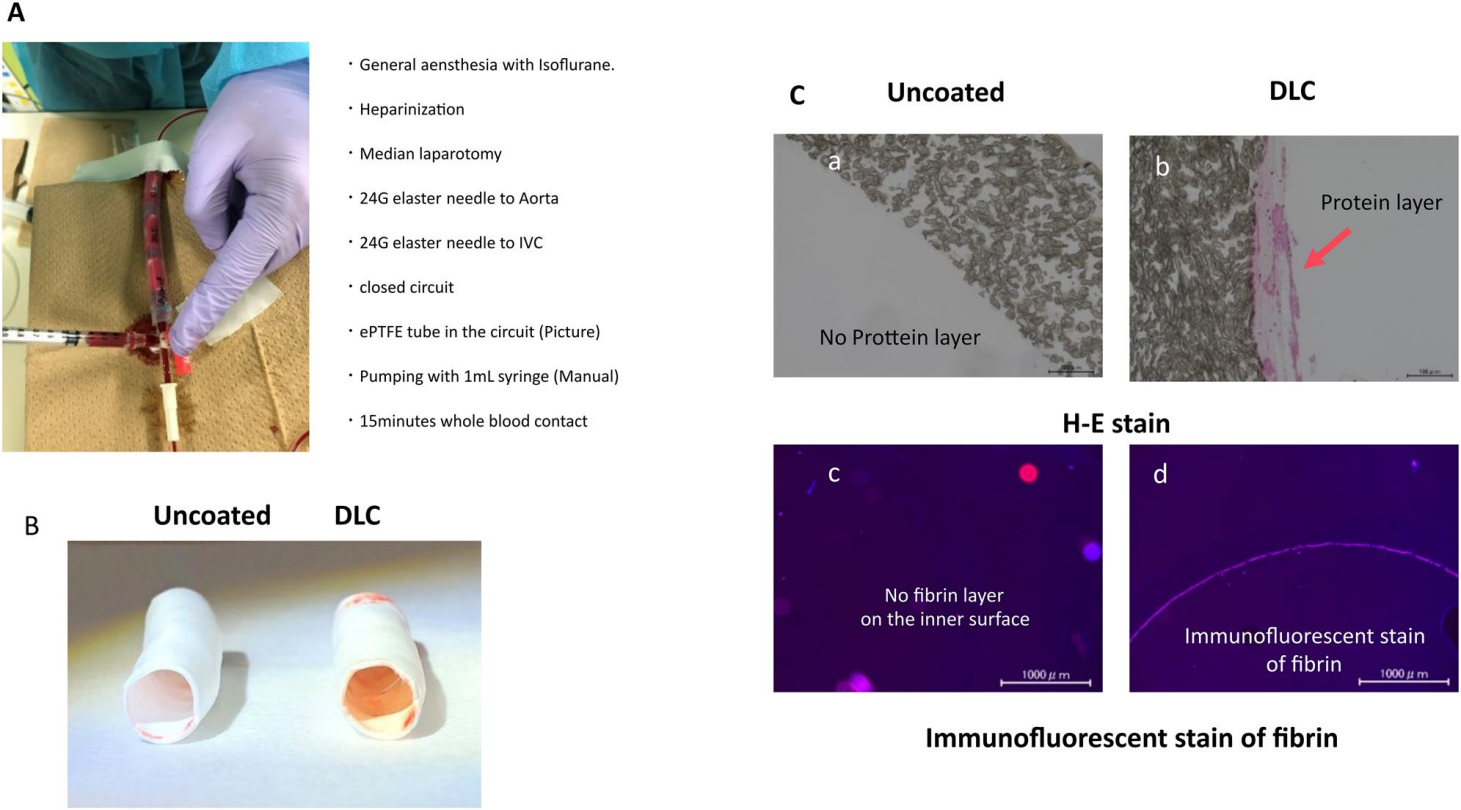
Results of in vivo whole blood contact tests using rats. (**A**) The circuit for the examination. (**B**) Macroscopic appearance of the ePTFE grafts after examination. (**C**) Existence of fibrin layer on the surface of ePTFE grafts. H-E; hematoxylin eosin.

Figure 5 depicts the circuit for the in vivo whole blood contact test in rats (Fig. 5A) and the results obtained. There was negligible blood in, both, the DLC-coated graft and uncoated graft (Fig. 5B). However, there was a thin fibrin layer on the inner surface of DLC-coated graft (Fig. 5C), similar to the data obtained in in vitro human blood contact test.

#### Whole blood contact test with arterial or venous replacement in swine (non-survival implantation study to observe the macroscopic condition of ePTFE blood-contact surface in pulsatile or stagnant flow condition)

**Figure 6:**
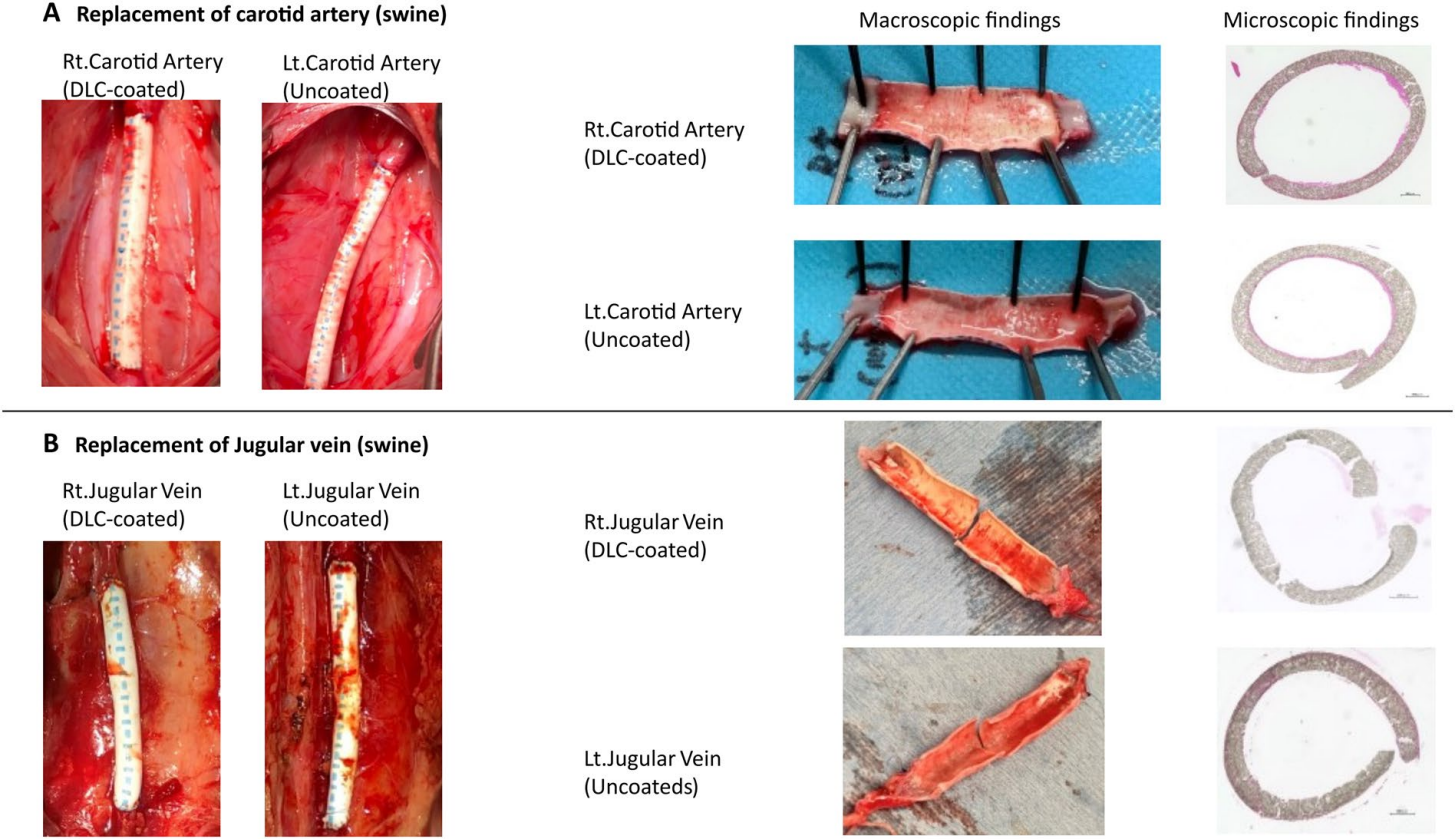
Results for 3 h whole blood contact tests using carotid artery and jugular vein replacement swine models. (**A**) Pictures of a whole blood contact tests with replacements of carotid arteries. (**B**) Pictures of a whole blood contact tests with replacements of jugular veins.

Figure 6 shows the results of the replacement of carotid arteries (Fig. 6A) and the replacement of jugular veins (Fig. 6B) with 4 mm ePTFE grafts. After 3 h in vivo blood flow contact, the grafts were harvested and opened longitudinally. The macroscopic appearance was similar in both DLC-coated and uncoated grafts, with a thin mucous coating and scarce blood clots in both the arterial and the venous replacements. Microscopic examination of hematoxylin–eosin stained sections showed the absence of blood clots, with very thin protein layer and few attached nucleated cells in all grafts. These results indicate that, with short term in vivo blood contact, uncoated and DLC-coated ePTFE grafts have high hemocompatibility with the same degree in both pulsatile blood flow condition and constant blood flow condition.

### Surgical implantation studies with animal survival

Table 1 shows results of all the animal survival studies. Each animal study was done in a consecutive manner. The data obtained are as follows.

**Table 1:** Results of all animal survival studies.

Animal species	No.	Procedure	Vessels	ePTFE type	Survival (weeks)	Patency	Anastomotic stenosis
Rat	1	Aortic replacement using hand-made 1.5 mm ePTFE graft	Aorta	Uncoated	1	Patent	Mild
	2			Uncoated	1	Patent	Moderate
	3			Uncoated	0	Occluded	No
	4			Uncoated	0	Occluded	No
	5			DLC	0	Occluded	No
	6			DLC	1	Patent	Mild
	7			DLC	0	Occluded	No
	8			DLC	1	Patent	Mild
	9			DLC	1	Patent	Mild
	10			Uncoated	1	Patent	Mild
	11			DLC	1	Patent	Mild
Goat	1	Arteriovenous graft using 4 mm ePTFE graft	Rt.CA—Rt.JV	Uncoated	8	Patent	Very severe
			Lt.CA—Lt.JV	DLC		Patent	Very severe
	2		Rt.CA—Rt.JV	DLC	8	Patent	Very severe
			Lt.CA—Lt.JV	Uncoated		Patent	Very severe
	3		Rt.CA—Rt.JV	Uncoated	8	Patent	Very severe
			Lt.CA—Lt.JV	DLC		Patent	Very severe
	4		Rt.CA—Rt.JV	DLC	8	Patent	Very severe
			Lt.CA—Lt.JV	Uncoated		Occluded	Occluded
	5		Rt.CA—Rt.JV	Uncoated	8	Patent	Very severe
			Lt.CA—Lt.JV	DLC		Patent	Very severe
	6		Rt.CA—Rt.JV	DLC	8	Patent	Very severe
			Lt.CA—Lt.JV	Uncoated		Patent	Very severe

CA, carotid artery; DLC, diamond-like carbon; ePTFE, expanded polytetrafluoroethylene; JV, jugular vein.

#### Aortic replacement in rats (small arterial bypass model with 7-days survival after surgery)

**Figure 7:**
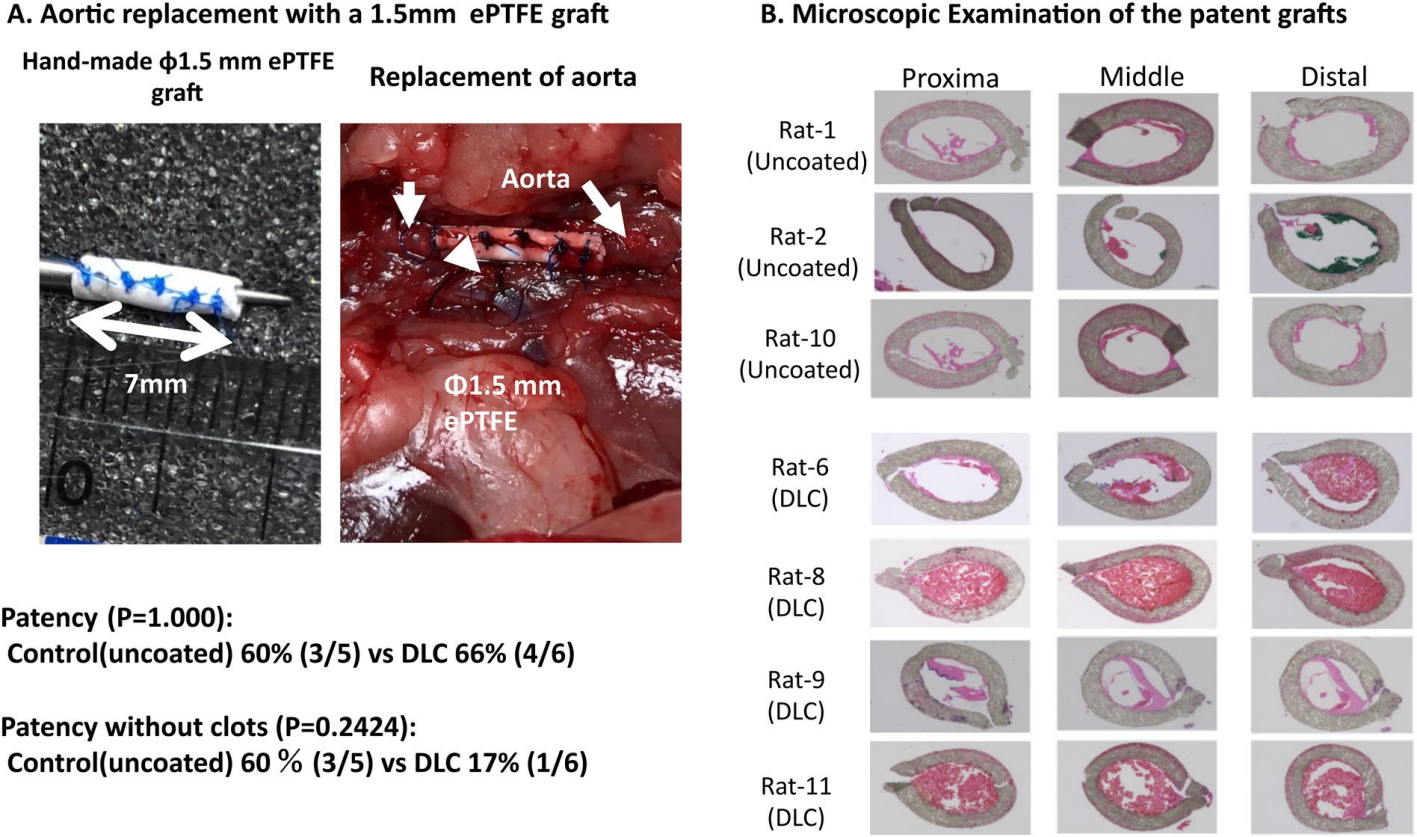
Results of aortic replacement in rats using handmade 1.5 mm DLC-coated and uncoated ePTFE grafts. (**A**) Macroscopic appearance of 1.5 mm ePTFE graft. (**B**) Microscopic appearance of the explanted grafts. The patency was comparable. However, DLC-coated ePTFE may tend to have more clot formation in the graft.

A hand-made 1.5 mm small ePTFE graft was implanted in each animal (Fig. 7A). Seven grafts were patent 7 days after surgery and four grafts were occluded due to thromboembolism (Table 1). All of the anastomotic sites were mildly stenotic (< 50% stenosis) except for 1 moderate stenosis (50%–74%) of the proximal anastomotic site in animal No. 2 in uncoated ePTFE group. No significant differences were observed in the patency rates (60% (3/5) in uncoated ePTFE and DLC-coated ePTFE (67% (4/6)) (*P* = 1.000). Histological analysis of 7 patent grafts showed definite thrombus in three of the four patent DLC-coated ePTFE grafts; in contrast, almost no thrombus was observed in all of the patent uncoated ePTFE grafts (Fig. 7B). However, there was no statistically significant difference (*P* = 0.2424) for the clot free patency in uncoated ePTFE (60% (3/5)), compared to that in DLC-coated ePTFE (17% (1/6)).

#### Arteriovenous graft in goats (AVG model with 8 weeks survival after surgery)

Graft patency was evaluated using an ultrasonic doppler blood flow meter before the explantation. Although there was very severe stenosis (difficult to identify the source by macroscopic observation) at all anastomotic sites, there were weak pulsatile blood flow sounds on the ultrasonic doppler blood flow meter in all the six DLC-coated ePTFE grafts and five of the six uncoated ePTFE grafts. In addition, saline could be flushed through the graft. Therefore, these 11 grafts were regarded as patent. There was no statistical difference in the patency rate between the DLC-coated and uncoated ePTFE graft (*P* = 1.000). Despite the existence of very severe stenosis at the anastomosis, all the patent ePTFE grafts were open without being filled with thrombus. This also indicates the presence of blood flow just before the harvest.

## Discussion

DLC was coated on the internal surface of ePTFE vascular grafts using a previously reported original chemical vapor deposition method. SEM analysis revealed that DLC-coating decreased roughness of the internal surface of the ePTFE grafts while maintaining its porous structure. In addition, *R*_*a*_ and *R*_*q*_ values were clearly decreased in DLC-coated ePTFE, which means DLC-coated ePTFE was smoother than uncoated ePTFE. Surface roughness is an important factor affecting hemocompatibility because a rougher surface exposes more surface area to blood, promoting more rapid coagulation^15^. Hence, the decreased surface roughness resulting from the DLC-coating can lead to improved hemocompatibility. In addition, there was no obvious micro-peeling of the DLC coating even after the anastomotic procedures, indicating sufficient durability of the DLC coating. We did not perform any mechanical loading tests to measure the stability of the DLC-coating used in this study; however, a tensile test was performed with ePTFE sheets coated with DLC created using argon gas. The results are shown in Supplementary Figure S2. The plasma processing time varied to produce DLC with different thicknesses. Accordingly, 5-, 20-, and 60-min argon plasma processing made 20, 100, and 300 nm DLC thicknesses, respectively. Then, we performed the tensile test with 80% stretch of the sheets and found that 20 nm, as opposed to 100 and 300 nm, DLC had no cracks and breaks of the fibril structure. This explains why the processing time applied herein was set to 5 min. Generally, DLC created using methane plasma is softer than that created using argon plasma. Therefore, the DLC used in this study has enough mechanical loading stability for medical use, such as in catheters and vascular grafts.

The increased hydrophilicity of DLC-coated ePTFE sheet surface was confirmed with significantly decreased water contact angle of the DLC surface. This phenomenon, along with the results of Raman spectrography, also indicate that DLC was successfully coated to the surface. In addition, the range of the values of water contact angle was very narrow and the standard deviation was very small. This fact indicates that the small numbers of samples in statistical analysis for the protein adsorption test and platelet adhesion test can be justified because the quality of each surface was homogenous in both DLC-coated and uncoated ePTFE. DLC can impart long-lasting hydrophilicity to the coated surface due to the semi-permanent durability^16^. Increased hydrophilicity could have a positive effect on ePTFE grafts, as hydrophilic surfaces are reported to be less thrombogenic than hydrophobic surfaces^17^.

The interaction of biomaterials and blood is initiated with the adsorption of plasma proteins, such as albumin and fibrinogen, on the material’s surfaces. SDS-PAGE examination using human whole blood showed that both types of grafts had similar electrophoretic properties. However, the bands for albumin, fibrinogen, heavy IgG, and light IgG tended to be clearer in DLC-coated ePTFE grafts than those in uncoated ePTFE grafts. The albumin and fibrinogen adsorptions were also significantly increased, based on the adsorption test for these proteins. Generally, it is said that albumin has a high affinity to hydrophilic surfaces, and fibrinogen has a high affinity to hydrophobic surfaces^18^. However, in this study, both albumin and fibrinogen adsorption were increased in DLC-coated ePTFE despite the increased hydrophilicity of the DLC surface. A previous study reported that both albumin and fibrinogen adsorption increased with DLC coating from the baseline of substrates^19,20^. Jones et al*.*^21^ compared proteins in which the most hydrophilic surfaces had the maximum fibrinogen adsorption with titanium. Liao et al.^22^ reported that sp^2^/sp^3^ ratio in DLC coating considerably influenced biocompatibility. Presumably, a change in hydrophilicity does not solely determine protein adsorption. The sp^2^/sp^3^ ratio, hydrogen content, and oxygen content of DLC, as well as the properties of the substrate itself, are thought to affect protein adsorption after DLC coating. In addition, preferential albumin adsorption is known to inhibit platelet adhesion and activation^23,24^, while preferential fibrinogen adsorption promotes platelet adhesion and activation^9^. Therefore DLC-coating exhibited both positive and negative aspects of protein adsorption on the surface hemocompatibility. However, in this study, the albumin to fibrinogen adsorption ratio was increased from 0.218 to an average of 0.231. This might explain why DLC-coated ePTFE maintains comparable hemocompatibility despite the increased fibrinogen adsorption, since a higher albumin to fibrinogen ratio results in better hemocompatibility^25^.

A high degree of platelet adhesion and activation can lead to thrombus formation. Thus, platelet surface adhesion and activation are the most important factors to assess hemocompatibility of a biomaterial. In this study, DLC reduced platelet adhesion and, platelets on DLC-coated grafts retained their round shape and were less likely to show pseudopodia formation, which indicates less activation of the platelets^26^. These phenomena are positive aspects on the surface hemocompatibility.

Whole blood contact tests were performed using human blood in vitro and blood of rats and swine in vivo. Blood clot formation was scarce and comparable between uncoated ePTFE and DLC-coated ePTFE in all the three tests, although there was a thin fibrin layer after in vitro human (static condition) and in vivo rat (flow condition) whole blood contact tests, which is consistent with the results of fibrinogen adsorption test and SDS-PAGE. However, increased fibrinogen attachment does not always imply less surface hemocompatibility as described in a previous report^27^. The results of the swine model in this study indicate that the short term hemocompatibility in both pulsatile and constant blood flow condition is comparable between DLC-coated and uncoated ePTFE surface.

The patency in aortic replacement in rats and AVG in goats were comparable between DLC-coated and uncoated ePTFE grafts. There was negligible blood clot formation in AVG using 4 mm ePTFE. However, in aortic replacement rat model, using 1.5 mm hand-made ePTFE graft, three of the four patent DLC coated ePTFE grafts had considerable blood clots. This is probably because increased fibrinogen adsorption on DLC-coated ePTFE surface negates the other hemocompatibility-based beneficial effects of DLC, such as increase hydrophilicity, surface smoothness, and albumin adsorption and decreased platelet adhesion. In AVG goat model, severe anastomotic stenosis was a critical issue to be addressed for evaluating the clinical usability of DLC-coated ePTFE grafts in mid-to-long term survival studies; this animal model required further improvements. We tried a few similar experiments using swine, but the situation did not change, and granulation formation at the anastomotic site was a problem, as reported in existing reports^28^. However, this phenomenon did not affect the interpretation regarding hemocompatibility. Thus, with results of these animal implantation studies, we can conclude that the function of DLC-coated ePTFE is comparable to that of an ePTFE surface using 4 mm ePTFE graft; however, the unfavorable aspect of increased fibrinogen attachment may increase clot formation in ePTFE grafts with considerably smaller diameter.

In summary, DLC-coating has high hemocompatibility at the ePTFE level, however; it failed to improve the patency of very small diameter vascular grafts. DLC coating will be beneficial in economically providing hemocompatibility comparable to the ePTFE level in less hemocompatible materials. Fortunately, there are several ways of modifying DLC surfaces^9,29^ which are generally easier and more economical than coating with organic materials. We intend to research further to achieve better hemocompatibility with DLC.

In the aortic replacement study using rats, we used a handmade 1.5 mm diameter ePTFE vascular graft due to non-availability of a flaw-free graft with this diameter. The quality of the suture line on the graft may influence the results of this experiment. However, the reproducible results were observed in this study. There is a small possibility that this limitation may severely affect the study conclusion.

In conclusion, the DLC-coated ePTFE graft showed comparable hemocompatibility using in vitro and in vivo tests. Therefore, DLC coating can effectively be used to achieve hemocompatibility similar to ePTFE among resin fabrics with hemocompatibilities lesser than that of ePTFE.

## Methods

### Ethical approval

Blood sample collection from human volunteers was approved by the Ethics Committee at Okayama University and conducted in accordance with the Declaration of Helsinki and regulations for human research in Japan (Ethical Guidelines for Medical and Biological Research Involving Human Subjects). They gave their written informed consent to participate in the study.

All animal experiments described in this study were approved by the Animal Care and Use Committee at Okayama University. All animals were cared in strict accordance with the recommendations in the guidelines for the care and use of laboratory animals issued by the National Institutes of Health, the ARRIVE guidelines, and regulations for animals in Japan (Standards relating to the Care and Keeping and Reducing Pain of Laboratory Animals. (Notice of the Ministry of the Environment No. 88 of 2006). Latest revision: Notice of the Ministry of the Environment No. 84 of 2013).

### DLC coating on the internal surface of ePTFE grafts and both sides of ePTFE sheets

The internal surfaces of ePTFE artificial vascular grafts (thin wall artificial vascular graft; W.L. Gore & Associates G.K., Tokyo, Japan) and both sides of ePTFE sheets (0.1 mm thickness artificial pericardial membrane, W.L. Gore & Associates G.K., Tokyo, Japan) were coated with DLC using an alternating current high-voltage radiofrequency methane plasma chemical vapor deposition system. The deposition conditions for ePTFE sheets were as follows: ultimate vacuum pressure, 7.0 × 10^−3^ Pa; process pressure, 5 Pa; CH_4_ gas introduced at 10 sccm using a mass flow controller; RF output power, 70 W; and processing time, 5 min. Coating with DLC was confirmed using Ramen spectroscopy. Details on the ePTFE graft deposition system and methods of Ramen spectroscopy have been reported previously^14^. Grafts and sheets were sterilized using ethylene oxide prior to each experiment.

### Measurement of the water contact angle

ePTFE sheet was used because plane surface is required for the accurate measurement. The hydrophilicity of uncoated (n = 10) and DLC-coated ePTFE sheets (n = 10) was evaluated by measuring the static contact angle between a 2 µL deionized water drop and the surface of each sheet using DropMaster500 (Kyowa Interface Science, Saitama, Japan). The water contact angle was averaged for six samples from each group for comparison.

### SEM analysis

Uncoated and DLC-coated ePTFE grafts or sheets were cut into 8 mm × 6 mm segments for SEM analysis of surface characteristics. Specimens were processed using the standard osmium tetroxide method after fixation, dehydration, and critical point drying. Dried specimens were affixed to specimen mounts with double-sided tape. Specimens were coated with 15 nm of gold–palladium and examined at 5.0 kV using a HITACHI S-4800 SEM (Hitachi High-Tech Corporation, Tokyo, Japan).

To determine whether the DLC coating peeled off after anastomotic maneuver, two DLC-coated grafts were anastomosed to each other using a 6–0 polypropylene running suture. The anastomotic site was then observed using SEM.

### Quantitative evaluation of surface roughness of ePTFE sheets

Pictures of ePTFE sheets obtained from SEM were used because plane surface was required for this analysis. The surface roughness/smoothness of ePTFE sheets were quantitatively evaluated using the following metrics:

1 $$R_{a} = \frac{1}{n}\mathop \sum \limits_{i = 1}^{n} \left| {p_{i} - \overline{p}} \right|$$

2 $$R_{q} = \sqrt {\frac{1}{n}\mathop \sum \limits_{i = 1}^{n} \left( {p_{i} - \overline{p}} \right)^{2} }$$

where *R*_*a*_ is the arithmetic mean roughness, *R*_*q*_ is the root-mean-square roughness, n is the number of pixel data, *p*_*i*_ is pixel value, and $\overline{p}$ is the mean value of the pixel data. The smaller *R*_a_ or *R*_*q*_ indicates smoother surface: *R*_a_ or *R*_*q*_ = 0 means completely smooth (flat) surface. Since the image data usually include noise, and the pixel values are expected to spatially fluctuate. To remove the noise, Detrending method called trend filtering^30^ was used for data preprocessing.

### Protein adsorption tests

Protein adsorption tests were performed by treating ePTFE grafts with human serum albumin and fibrinogen solutions. Both human albumin (12.5 g/50 mL human albumin; Japan Blood Product Organization, Tokyo, Japan) and human fibrinogen (fibrinogen from human plasma; 50–70% protein [≥ 80% of protein is clottable]; Sigma-Aldrich Co. LLC., Burlington, MA, USA) were dissolved separately in phosphate-buffered saline (PBS) (pH 7.4) to concentrations of 1 mg/mL and 2.5 mg/mL, respectively. The samples (1.5 cm long ePTFE grafts) were initially rinsed with PBS and immersed in the protein solutions at 37 °C for 60 min. After incubation, the samples were carefully washed with distilled water, treated with 5% sodium dodecyl sulfate (SDS), and rinsed overnight at 37 °C. Following this, ultrasonication was performed for 10 min for complete protein desorption from the samples. The concentrations of albumin and fibrinogen in the resultant solutions were measured using a Micro BCA™ Protein Assay Kit (Thermo Fisher Scientific K.K., Tokyo, Japan) according to the manufacturer’ s protocol and bovine serum albumin as a standard.

### Platelet adhesion test

Human whole blood (45 mL) was collected from three healthy volunteers who were not under any medication for at least two weeks after obtaining informed consent. Each blood sample was mixed with 5 mL of sodium citrate and centrifuged at 180 × g for 15 min to obtain platelet-rich plasma (PRP). Platelet-poor plasma (PPP) was obtained by centrifugation of PRP at 2000 × *g* for 10 min. Subsequently, PRP and PPP were combined in suitable proportions to achieve a platelet concentration of 3.0 × 10^5^/µL (adjusted PRP). After washing the ePTFE sheet and graft samples with PBS, they were immersed in 1.5 mL of adjusted each PRP samples and incubated at 37 °C for 30 min in an atmosphere containing 5% CO_2_ gas. After incubation, the samples were washed carefully with PBS and fixed in 1.5 mL of 25% glutaraldehyde for 60 min at room temperature, followed by careful washing with PBS. After rinsing, the samples were observed using fluorescence microscopy (All-in-one Fluorescence Microscope BZ-X700, KEYENCE, Osaka, Japan) and glutardialdehyde-induced fluorescence technique reported by Frank et al.^31^; the platelet-covered area per field of view (in %) was calculated with 15 fields of view from three samples in each group. The samples were also dried at their critical point, and the morphology of adherent platelets was observed using SEM.

### In vitro human whole blood contact test (SDS-PAGE and immunofluorescent staining of fibrin)

Human whole blood (45 mL) was collected from two healthy volunteers who were not under any medication for at least two weeks after obtaining informed consent. The blood was mixed with 5 mL of 3.2% sodium citrate. The samples (1.5 cm long ePTFE grafts) were initially rinsed with a PBS solution and immersed in the whole blood at 37 °C for 60 min on a shaking machine. After incubation, the samples were carefully washed in distilled water, treated with 5% SDS, and rinsed overnight at 37 °C. Following this, ultrasonication was performed for 10 min to completely desorb the proteins from the samples. The samples were preserved at −80 °C for sodium dodecyl sulfate–polyacrylamide gel electrophoresis (SDS-PAGE) analysis.

For SDS-PAGE analysis, 340 µL of this sample was put into a 2.0 mL Low Protein Binding Microcentrifuge Tubes (Thermo Fisher Scientific K.K., Tokyo, Japan), with 1.36 mL of acetone and was left undisturbed overnight at −30 °C. Further, it was centrifuged at 20,000 × *g* at 4 °C for 10 min, the supernatant was disposed, and the tubes were dried. Twenty microliter of SDS-PAGE sample buffer, composed of 325 µL water + 125 µL NuPAGE LDS sample buffer (4×) (Thermo Fisher Scientific K.K., Tokyo, Japan) + 50 µL NuPAGE reducing agent (10×) (Thermo Fisher Scientific K.K., Tokyo, Japan) was added to the tubes and stirred. The sample was heated at 95 °C for 5 min, followed by cooling for 5 min, to obtain the SDS-PAGE loading sample. The proteins (20 µg) were resolved via SDS-PAGE and transferred to a NuPAGE Bis–Tris Protein Gel (Thermo Fisher Scientific K.K., Tokyo, Japan). Bands were stained using SYPRO™ Ruby Protein Gel Stain (Thermo Fisher Scientific K.K., Tokyo, Japan) and visualized. The samples obtained from this study were also used for immunofluorescent staining of fibrin.

### In vivo whole blood contact tests

#### In vivo rat whole blood contact test

Sprague–Dawley rats (Japan SLC, Inc., Shizuoka, Japan) were anesthetized by the inhalation of 5% isoflurane and maintained with 2–3% isoflurane using a hand-made mask. The aorta and inferior-vena-cava (IVC) were exposed via a laparotomy. Heparin 100 (IU/100 g body weight) was administered and 24G elaster needles were inserted into the aorta and IVC. A handmade small circuit, composed of intravenous lines, a three-way-stopcock, and a 5 mm vinyl tube containing the ePTFE grafts, was connected to the elaster needles. Blood was circulated by manual pumping for 15 min using a 1 mL syringe. After the 15 min blood contact, the ePTFE grafts were macroscopically observed, rinsed gently, and preserved for histological examination. The samples obtained from this study were also used for immunofluorescent staining of fibrin.

#### Whole blood contact test with arterial or venous replacement in swine (non-survival implantation study to determine macroscopic condition of ePTFE blood-contact surface in pulsatile or stagnant flow condition)

Two swine weighing 30 kg (National Federation of Agricultural Cooperative Association, Tokyo, Japan) were used. Anesthesia was induced with an intramuscular injection of atropine sulphate (0.05 mg/kg), ketamine hydrochloride (5 mg/kg), and medetomidine hydrochloride (0.08 mg/kg), maintained with 1–2% isoflurane. Heparin (5 mL) was administered intravenously. A DLC-coated 4 mm ePTFE and uncoated ePTFE vascular grafts were implanted to the right and left carotid arteries or the right and left jugular veins using 7–0 polypropylene running sutures, respectively. Following this, doppler ultrasound examination was performed to ensure there is no blood flow acceleration around the anastomosis sites (< 1.0 m/s). Blood was passed through the grafts for 3 h. After harvesting the grafts, they were opened longitudinally and macroscopic pictures of the blood-contacted surface of the ePTFE grafts were taken. The graft was fixed in 10% neutral buffered formalin for microscopic tissue examination. The animals were euthanized using an intravenous injection of potassium chloride.

#### Microscopic examination after whole blood contact test

Grafts were fixed in 10% neutral buffered formalin, cut into 3 mm-thick cross sections and embedded in paraffin. Cross Sects. (5 µm thick) of the grafts were stained with hematoxylin–eosin. Immunofluorescent staining of fibrin was also performed with a fibrin antibody [UC45] (GTX19079, Gene Tex, California, USA) and an immunoglobulin (Ig)G (H + L) goat anti-mouse secondary antibody (Alexa Fluor 647, Thermo Fisher Scientific, Massachusetts, USA) using samples from in vitro human and in vivo rat whole blood contact tests. The nuclear counterstain was performed using 4′,6-diamidino-2-phenylindole.

### Surgical implantation studies with animal survival

#### Aortic replacement in rats (small arterial bypass model with 7 days survival after surgery)

Eleven consecutive male Sprangue-Dawley rats (Japan SLC, Inc., Shizuoka, Japan) were used for this study. All animals underwent aortic replacement using hand-made 1.5 mm-ePTFE graft (uncoated ePTFE; n = 5, DLC-coated ePTFE; n = 6). The hand-made 1.5 mm graft was created from 4 mm ePTFE vascular grafts (thin wall artificial vascular graft; W.L. Gore & Associates G.K., Tokyo, Japan). After opening and trimming, it was wrapped around 1.5 mm Hegar dilator and sutured using a 7–0 polypropylene interrupted sutures.

A midline laparotomy was performed, under mask anesthesia, with 2% isoflurane, and the infra-renal aorta was dissected. Heparin sulphate (0.3 mL) was injected into the infra-renal aorta with a 27G needle, and the aorta was clamped just below the renal arteries. The aorta was replaced with a hand-made 1.5 mm ePTFE vascular graft (7 mm length) using 7–0 polypropylene interrupted sutures. After closing the laparotomy, the animal was awakened and returned to the cage. Feeding was resumed on the first postoperative day. All animals were housed for 7 days with humane care. These grafts were explanted after flushing saline and 10% neutral formalin into the graft and aorta under the same general anaesthesia condition as described above. After explanting the graft, the animals were euthanized by intravenous injection of potassium chloride. The obtained tissue and graft were fixed in 10% neutral buffered formalin for microscopic tissue examination.

### AVG in goats (AVG model with 8 weeks survival after surgery)

Six consecutive adult male goats weighing 32–54 kg (National Federation of Agricultural Cooperative Association, Tokyo, Japan) were used for this study. All animals underwent bilateral straight AVG with 4 mm ePTFE grafts (4 mm diameter thin wall artificial vascular graft; W.L. Gore & Associates G.K., Tokyo, Japan) using common carotid artery and ipsilateral external jugular vein under sterile conditions. From the day following operation, oral aspirin (100 mg) was administered daily to each goat. Atropine sulphate (0.05 mg/kg) was administered by intramuscular injection as premedication. All animals were sedated by an intramuscular injection of a liquid mixture of ketamine hydrochloride (5 mg/kg) and medetomidine hydrochloride (0.08 mg/kg) and anesthetized by the inhalation of 5% isoflurane. After intubation, anesthesia was maintained with 2–3% isoflurane using a ventilator. An intravenous infusion of lactated Ringer’s solution was maintained at a speed of 10 ml/kg/h throughout the procedure. The animals were placed in the supine position and the bilateral external jugular veins and common carotid arteries were exposed. Further, intravenous 100 IU/kg heparin was administered, followed by additional injection of 50 IU/kg every 60 min. An uncoated ePTFE graft was applied as a control on one side, and a DLC-coated ePTFE graft was applied on the contralateral side. All AVGs were prepared by standard end-to-side vascular anastomoses using 7–0 polypropylene running sutures. After each anastomosis was finalized, blood flow was restored, and a palpable thrill was confirmed. Animals were monitored postoperatively until they were able to breathe spontaneously and move to an upright position. Feeding was resumed on the first postoperative day. All animals were housed for 8 weeks with humane care. Graft patency was confirmed using an ultrasonic doppler blood flow meter on the first, second, fourth, and eighth week post-operation. After 8 weeks survival, all grafts were explanted under the same general anesthesia. Graft patency was evaluated by the detection of pulsatile blood flow in the external jugular vein near the graft-vein anastomosis using an ultrasonic doppler blood flow meter. After flushing saline and 10% neutral formalin into the graft, the common carotid artery, the external jugular vein, and the AVGs were removed and fixed en bloc without their dissection. The tissue was fixed with 10% neutral buffered formalin for microscopy. After explantation, the animals were euthanized by intravenous injection of potassium chloride.

### Microscopic observation of the harvested ePTFE grafts

All of the explanted grafts were stained with hematoxylin–eosin (H-E) to visualize the internal surface condition of the grafts.

### Statistical analyses

All statistical analyses were performed using IBM SPSS software, version 19.0.0 (SPSS Inc., Chicago, Illinois). Shapiro–Wilk and Kolmogorov–Smirnov tests were used to confirm the normality of the continuous data. The results of these tests are provided in Supplementary Table S1. If either of these tests were passed in DLC-coated and uncoated groups, unpaired *t*-test was used to analyze differences of continuous data between the two groups. Otherwise, Mann–Whitney U test was carried out. Fisher’s exact test was used to analyze differences of categorical data between the two groups. All statistical analyses were performed at a significance level of 5%. Continuous values are represented by mean and standard deviation.

## Supplementary Information

Supplementary Information 1.

Supplementary Information 2.

Supplementary Information 3.

## Data Availability

The datasets generated and/or analyzed during the current study are available from the corresponding author on reasonable request.

## Abbreviations

AVG: Arteriovenous bypass
DLC: Diamond-like carbon
ePTFE: Expanded polytetrafluoroethylene
IVC: Inferior vena cava
SEM: Scanning electron microscopy
SDS-PAGE: Sodium dodecyl sulfate–polyacrylamide gel electrophoresis (SDS-PAGE)
