# Supplementary material for: Comprehensive hemocompatibility analysis on the application of diamond-like carbon to ePTFE artificial vascular prosthesis
Source: Sci Rep. 2023 May 24;13:8386. doi: 10.1038/s41598-023-35594-7 (PMC10209052; doi:10.1038/s41598-023-35594-7)

Supplementary Figure S1. The results of SDS-PAGE

This is the original pictures of gels/blots of SDS-PAGE as a sample file. This file includes some bands for attached protein on the surface of carboxylated DLC (O2-DLC). O2-DLC is not a topic of this manuscript, therefore we excluded bands of O2-DLC for Figure 4 of this manuscript. Band 1 is the band of the marker in both set 1 and set 2.

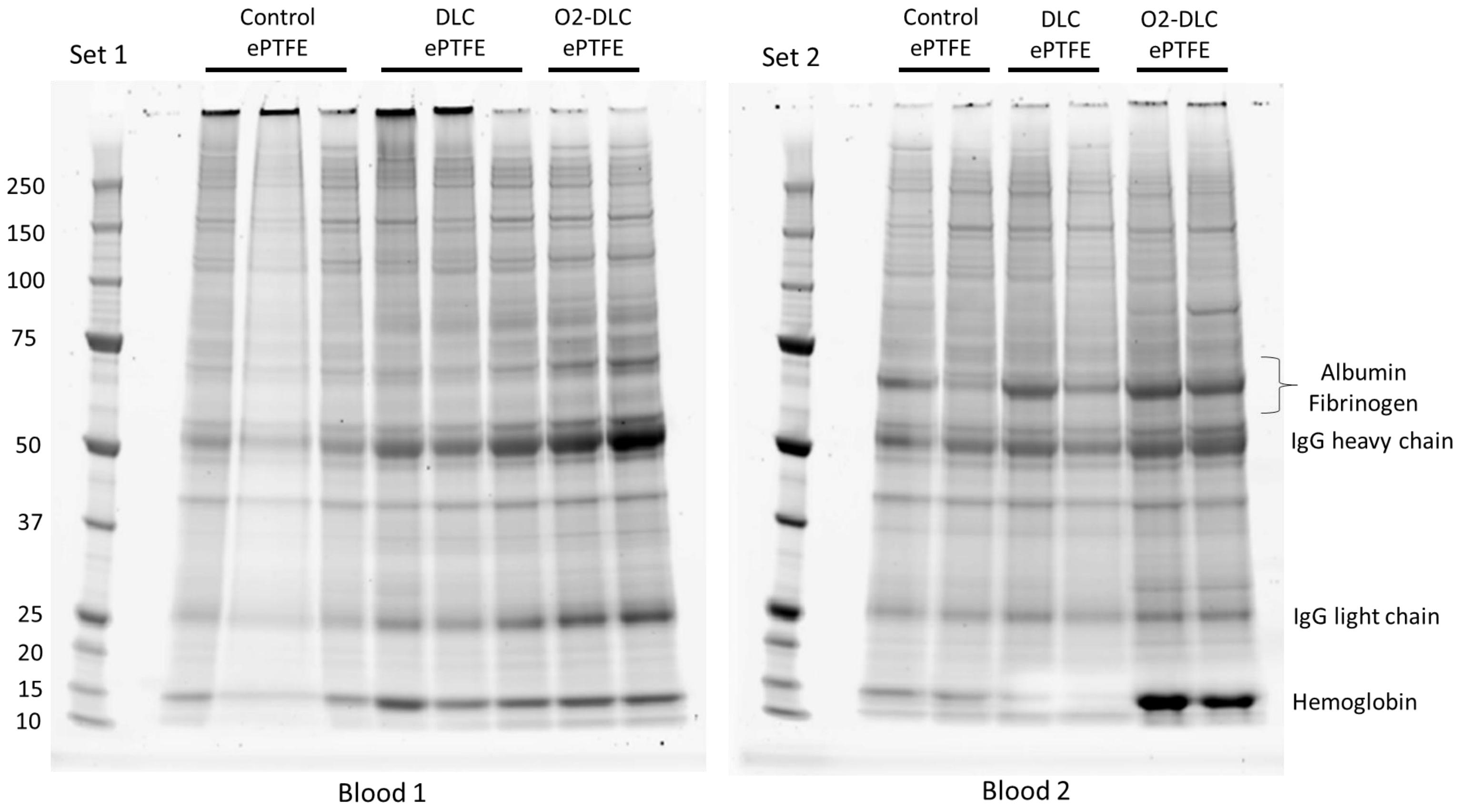

Supplement: Supplementary file 1 — Supplementary Information 1. [file 41598_2023_35594_MOESM1_ESM.pdf]
