# Supplementary material for: Comprehensive hemocompatibility analysis on the application of diamond-like carbon to ePTFE artificial vascular prosthesis
Source: Sci Rep. 2023 May 24;13:8386. doi: 10.1038/s41598-023-35594-7 (PMC10209052; doi:10.1038/s41598-023-35594-7)
Supplement: Supplementary file 2 — Supplementary Information 2. [file 41598_2023_35594_MOESM2_ESM.pdf]

Supplemental Figure S1. Methods of Tensile Test.

A tensile test of ePTFE sheet (0.1 mm thickness) was performed with Load testing machine（IMADA Ltd., Aichi, JAPAN）. The pulling spead was 10mm/min. DLC was coated with argon plasma chemical Vapor deposition system. The deposition conditions for ePTFE sheets were as follows; voltage, 500V; argon gas introduction at 20ccm; ultimate vacuum pressure, 0.2Pa. The processing time and resulted DLC thickness is as follows; 4 min, 20 nm; 20 min, 100 nm; 60 min, 200 nm.

Results of Tensile test

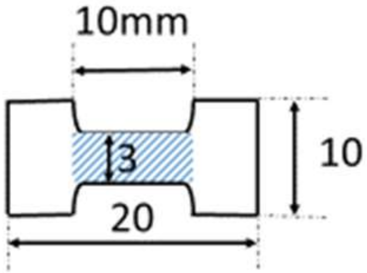

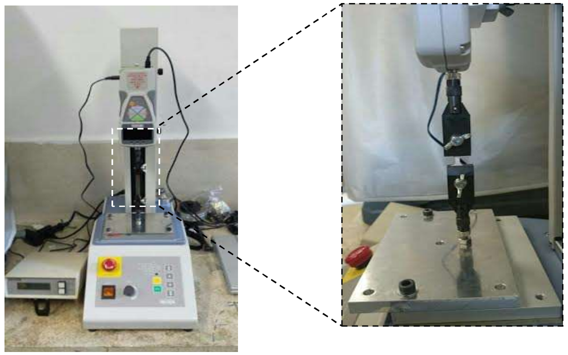

SEM Observation

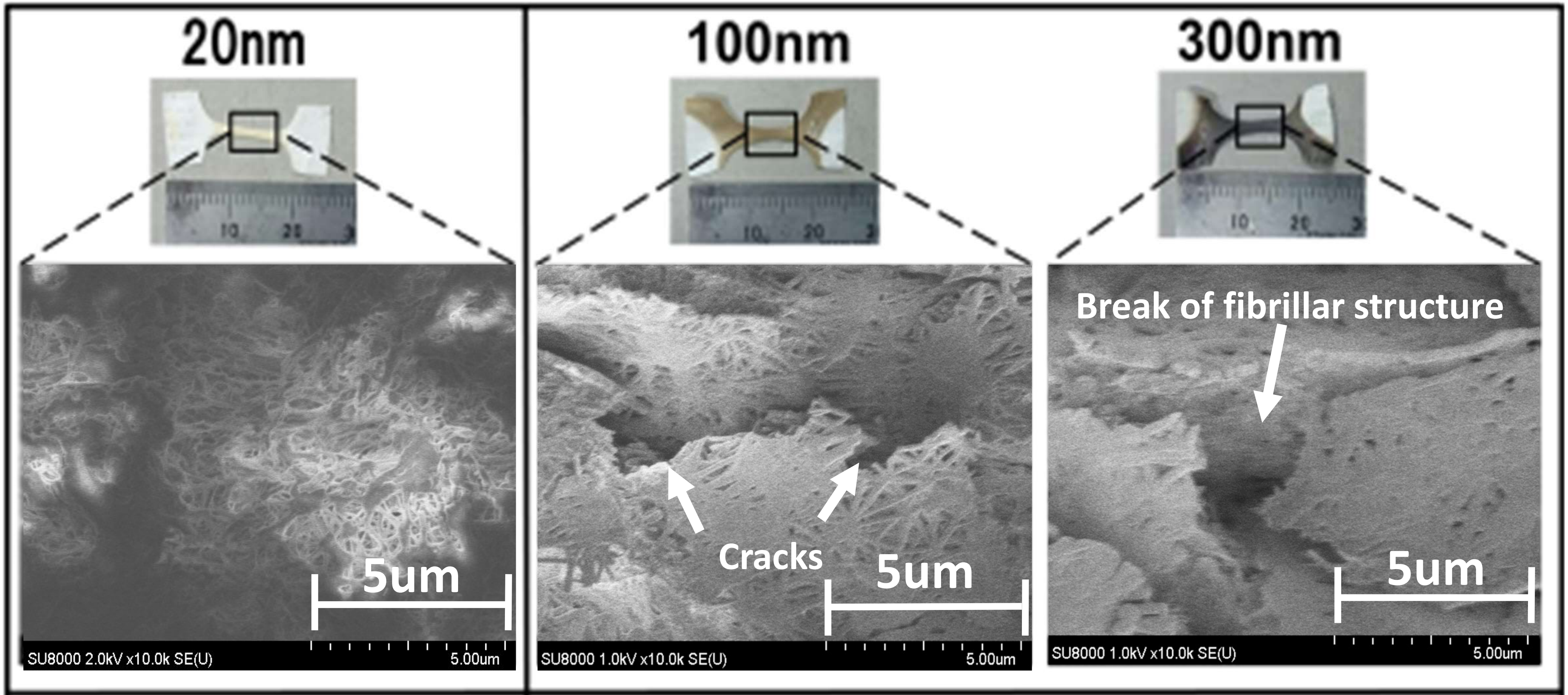

No cracks and breaks	There are cracks and breaks of ePTFE fibrillar structure

20 nm thickness DLC has better stability for mechanical loading than 100 nm thickness or 200 nm thickness DLC
