# Supplementary material for: Comprehensive hemocompatibility analysis on the application of diamond-like carbon to ePTFE artificial vascular prosthesis
Source: Sci Rep. 2023 May 24;13:8386. doi: 10.1038/s41598-023-35594-7 (PMC10209052; doi:10.1038/s41598-023-35594-7)
Supplement: Supplementary file 3 — Supplementary Information 3. [file 41598_2023_35594_MOESM3_ESM.pdf]

Supplemental Table S1. Results of Shapiro-Wilk test and Kolmogorov-Smirnov test.

Shapiro-Wilk test

	Control	DLC
Albumin test (Graft)	Passed (P=0.8012)	Not (P=0.0162)
Fibrinogen test (Graft)	Passed (P=0.5443)	Passed (P=0.4088)
Platelet test (Sheet)	Passed (P=0.638)	Not (P=0.0244)
Platelet test (Graft)	Not (P=0.0002)	Passed (P=0.181)
Water contact angle test (Sheet)	Passed (P=0.0563)	Passed (P=0.5031)

Kolmogorov-Smirnov test

	Control	DLC
Albumin test (Graft)	Passed (P>0.1000)	Not (P=0.0043)
Fibrinogen test (Graft)	Passed (P>0.100)	Passed (P>0.100)
Platelet test (Sheet)	Passed (P>0.100)	Passed (P=0.0774)
Platelet test (Graft)	Passed (P>0.100)	Passed (P>0.100)
Water contact angle test (Sheet)	Not (P=0.0318)	Passed (P>0.100)
